# Separating individual contributions of major Siberian rivers in the Transpolar Drift of the Arctic Ocean

**DOI:** 10.1038/s41598-021-86948-y

**Published:** 2021-04-15

**Authors:** Ronja Paffrath, Georgi Laukert, Dorothea Bauch, Michiel Rutgers van der Loeff, Katharina Pahnke

**Affiliations:** 1 Marine Isotope Geochemistry, Institute for Chemistry and Biology of the Marine Environment (ICBM), University of Oldenburg, Carl-von-Ossietzky-Str. 9-11, 26129 Oldenburg, Germany; 2 GEOMAR Helmholtz Centre for Ocean Research Kiel, Wischhofstr. 1-3, 24148 Kiel, Germany; 3 Leibniz-Labor, University of Kiel (CAU), Max-Eyth-Str. 11-13, 24118 Kiel, Germany; 4 Alfred Wegener Institute, Helmholtz Centre for Polar and Marine Research, Am Handelshafen 12, 27570 Bremerhaven, Germany

**Keywords:** Marine chemistry, Ocean sciences

## Abstract

The Siberian rivers supply large amounts of freshwater and terrestrial derived material to the Arctic Ocean. Although riverine freshwater and constituents have been identified in the central Arctic Ocean, the individual contributions of the Siberian rivers to and their spatiotemporal distributions in the Transpolar Drift (TPD), the major wind-driven current in the Eurasian sector of the Arctic Ocean, are unknown. Determining the influence of individual Siberian rivers downstream the TPD, however, is critical to forecast responses in polar and sub-polar hydrography and biogeochemistry to the anticipated individual changes in river discharge and freshwater composition. Here, we identify the contributions from the largest Siberian river systems, the Lena and Yenisei/Ob, in the TPD using dissolved neodymium isotopes and rare earth element concentrations. We further demonstrate their vertical and lateral separation that is likely due to distinct temporal emplacements of Lena and Yenisei/Ob waters in the TPD as well as prior mixing of Yenisei/Ob water with ambient waters.

## Introduction

**Figure 1:**
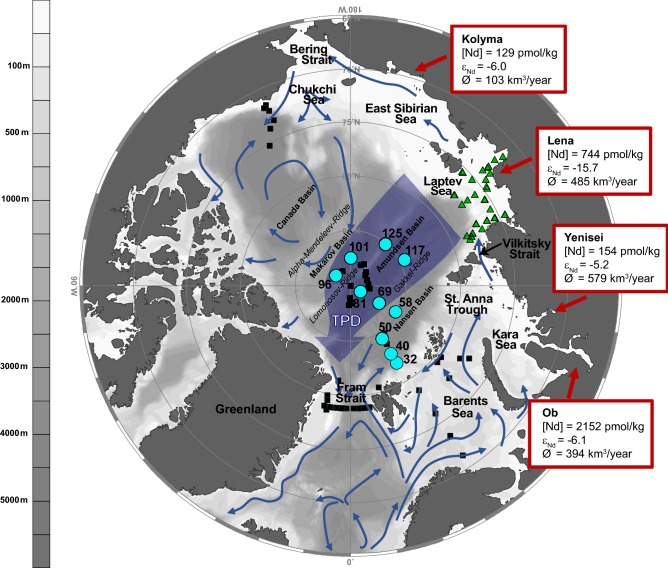
Map of the study region. Station locations along the GEOTRACES GN04 cruise transect in the central Arctic Ocean as turquoise dots. Black squares mark the locations of stations sampled for REEs^6,24,27,28,31,32^. Green triangles mark the location of data for the Laptev Shelf (for the Lena plume in 2013 and 2014 and the Vilkitsky Strait)^26^. Dark red arrows mark the areas of river input, the boxes provide river water Nd concentrations, ε_Nd_ composition^26,27^ and average annual river discharge^62^. Arrows show the schematic circulation of surface water^63^. TPD: Transpolar Drift. The map was produced using Ocean Data View^64^ (version 5.1.7, https://odv.awi.de/) and modified manually.

The Arctic Ocean is unique with respect to its high freshwater input from the Siberian and North American rivers (11% of global river discharge), which not only influences circulation, stratification and deep water formation in the high northern latitudes^1^ but also affects water column biogeochemistry and ecosystem functioning through addition of large amounts of river-borne macro- and micronutrients, as well as lithogenic elements to the open ocean^2–11^. The Siberian river influence, in particular, extends at least to the central Arctic Ocean, which is evident from elevated freshwater fractions and enhanced concentrations of river-borne nutrients and trace metals in the Transpolar Drift (TPD)^7,8,11^, a wind-driven sea ice and surface water current (1 to 5 km/day^11,12^) extending from the Siberian Shelf to the Fram Strait (see Fig. 1). The exact contributions of the individual Siberian rivers to the TPD and their spatiotemporal distribution within the TPD, however, have not been determined until now. The Siberian rivers extend over large areas and drain different regions influenced by highly distinct geologic, biologic and climatic conditions. Notably, the catchment areas of the Yenisei, Ob and Lena rivers, the largest Siberian river systems, are subject to no or continuous permafrost and entirely continuous permafrost, respectively^13,14^. This suggests that they will react differently to further warming, including irregular changes in river runoff and associated changes in riverine trace element, carbon and nutrient fluxes^15^. Predicted warming and associated changes in sea-ice extent, thickness and dynamics^16^ will also involve a significant reduction of sea-ice transport via the TPD^17^. In turn, this will affect upper water column stratification and circulation^18^ with adjustments of freshwater pathways and hence alterations in nutrient bioavailability and cycling, primary production and planktonic food webs. For evaluation of future climate impacts on sea-ice cover, stratification and circulation, as well as elemental fluxes and budgets and biological activity in the high northern latitudes, the spatial distribution and temporal variability of individual riverine contributions to and in the TPD need to be known.

Salinity, stable oxygen isotopes (δ^18^O) and nutrient relationships (e.g. N/P and/or PO_4_* (initial phosphate corrected for mineralization with oxygen, PO_4_^3−^ + O_2_/175 − 1.95 µmol/kg)) have been widely applied in the Arctic Ocean to determine the fractions of Atlantic, Pacific, meteoric water and sea-ice melt^3,19–21^. However, the distinction between Pacific and Atlantic water contributions based on nutrient relationships remains challenging and yields high uncertainties in the Pacific and Atlantic water fractions^3,22,23^, which inhibits reliable assessments of the water mass distribution in the upper water column of the open Arctic Ocean. Alternative assessments based on dissolved lithogenic trace elements and their isotopes, such as rare earth elements (REEs) and neodymium isotope ratios (^143^Nd/^144^Nd, expressed as ε_Nd_, see ‘Methods’), can help to disentangle contributing water masses and thus provide a better understanding of recent and upcoming changes in water mass distribution and mixing. Both REEs and ε_Nd_ are essentially biologically inactive provenance tracers and are therefore ideally suited to characterize the origin and transport pathways of waters contributing to the upper central Arctic Ocean and the TPD, given the highly varying REE concentrations ([REE]) and the distinct ε_Nd_ signatures of Atlantic (ε_Nd_ = − 11.7^6^) and Pacific (ε_Nd_ = − 5.5^24,25^) inflows, as well as the major Siberian rivers including the Lena river (ε_Nd_ = − 15.7^26^), and the Yenisei and Ob rivers (ε_Nd_ = − 5.2 and ε_Nd_ = − 6.1, respectively^27^). Previous studies based on REEs and ε_Nd_ have identified Atlantic water as the main contributing water mass to the Arctic Ocean and suggested its recirculation and outflow through the Fram Strait after cooling and partial mixing with Pacific and river water^6,24,28^. Strongly elevated [REE] and a wide range of Nd isotope compositions in Arctic surface waters have been related to inputs from Siberian rivers^24,27–30^, which are marked by variable but overall high [REE] and characteristic ε_Nd_ signatures (Fig. 1). Marginal Arctic regions such as the Laptev Sea, the Barents Sea and the Canadian Shelf areas were thoroughly investigated in the recent past for Nd isotope and REE behavior^26,31,32^. However, in the central Arctic Ocean, existing REE and Nd isotope data^24,27,28^ are sparse (Fig. 1 and Fig. S1) and their resolution in the upper water column is too poor to identify individual river water contributions. Here, we present a comprehensive dataset comprising dissolved ε_Nd_ and [REE] as well as δ^18^O from samples collected along the GEOTRACES transect GN04 (Fig. 1). Based on these tracer distributions we show that individual contributions of the major Siberian river systems, the Lena and Yenisei/Ob, are preserved along the TPD and largely do not mix during transport.

## Results

### Hydrography and water components based on salinity, δ^18^O and nutrients

The uppermost water column (0–200 m water depth) along the cruise track of PS94 (GEOTRACES transect GN04) comprises Polar Water (PW, including the Surface Mixed Layer and the Arctic Halocline; σ_θ_ < 27.70) characterized by a wide range of salinities (28–34.4), and underlying Atlantic and Arctic Atlantic Water (AW, AAW) with rather constant salinities approaching ~ 34.9 at 500 m water depth (see Rabe et al.^33^ and Table S1 for hydrographic data). The TPD in the central Arctic Ocean at the time of sampling was identified based on high CDOM (colored dissolved organic matter) fluorescence^8^ and is marked by low salinities due to river input. Outside the TPD, the PW is marked by higher salinities and lower nutrient and trace element concentrations^11^. Based on the CDOM definition, station 69 (Fig. 1) is considered to be outside the TPD. However, elevated [REE], a relatively radiogenic ε_Nd_ signal and a meteoric fraction of 2.5 to 7.1% suggest river influence from the surface down to 100 m water depth at station 69, which is in line with long-distance transport of Siberian river water via the TPD. We therefore consider stations 69–125 to be under the influence of the TPD, and stations 32–58 to be outside TPD influence.

The water mass analysis using salinity, δ^18^O and N/P ratios (see ‘Methods’ in the Supplementary Information) indicates a dominance of Atlantic water outside the TPD (i.e. at stations 32 to 58) and at all stations below 100 m depth. Meteoric contributions are highest (up to 20%) in the TPD at the surface at stations 81, 96 and 101 in line with low salinities, and decrease with depth, reaching 2–4% at 100 m water depth. Given that net in situ precipitation is expected to be small in volume and neglectable in REE content compared to riverine discharge^11^, the calculated meteoric fraction is used for the REE and ε_Nd_ interpretation as an equivalent to river water. Pacific water appears to be present in larger quantities of up to 84% at stations 96 and 101 in the Makarov Basin. However, the Pacific and meteoric fractions are moderately correlated (R^2^ = 0.73, not shown), suggesting a strong shelf or riverine component in our Pacific fraction calculated based on N/P ratios. Given the above observations and the generally high uncertainty associated with the calculations of the Pacific fraction (see Supplementary Information for details), we refrain from using the Pacific fraction and instead focus on the meteoric water fraction, which is based on salinity and δ^18^O only and hence essentially consistent between the different methods.

### Dissolved rare earth element and ε_Nd_ distributions

**Figure 2:**
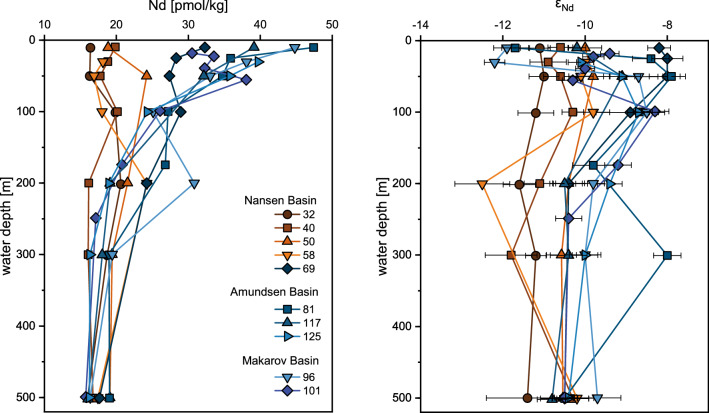
Vertical distribution of Nd concentration and Nd isotope composition. Water column profiles of Nd concentrations (left) and ε_Nd_ (right) for all stations along the transect. Stations outside the TPD are shown in brownish colors, stations within the TPD in blueish colors. Error bars are external errors for Nd concentrations (1SD, error bars usually smaller than the symbols) and propagated errors for ε_Nd_ (2SD).

In the low-salinity PW of the central Arctic Ocean with elevated meteoric fraction that marks TPD influence, [REE] are markedly elevated at the surface with [Nd] of up to 47.4 pmol/kg and [Er] of up to 13.5 pmol/kg (stations 69–125) and rapidly decrease towards ~ 300 m water depth (Fig. 2). In the higher salinity PW in the Nansen Basin outside the TPD (stations 32–58), where Atlantic water dominates, [REE] are lower between 16.2–24.2 pmol/kg for Nd and 4.7–7.1 pmol/kg for Er and rather constant with depth (Fig. 2, Table S1). The [REE] in these two areas converge below 200–300 m water depth, where the Atlantic water fraction is near 100% at all stations, to values of 17.0 ± 1.2 (1SD) pmol/kg Nd and 4.9 ± 0.2 (1SD) pmol/kg Er at 500 m water depth. The high [REE] in the upper water column (< 200 m) within the TPD correlate with the fraction of meteoric water (R^2^ = 0.49 and 0.65 for Nd and Er, respectively, p-value < 0.05 for both) and dissolved organic carbon (DOC^11^) (R^2^ = 0.78 and 0.88 for Nd and Er, respectively, p-value < 0.05 for both; Fig. S2), suggesting substantial terrestrial input of dissolved REEs via the Siberian rivers and transport of their discharge via the TPD.

**Figure 3:**
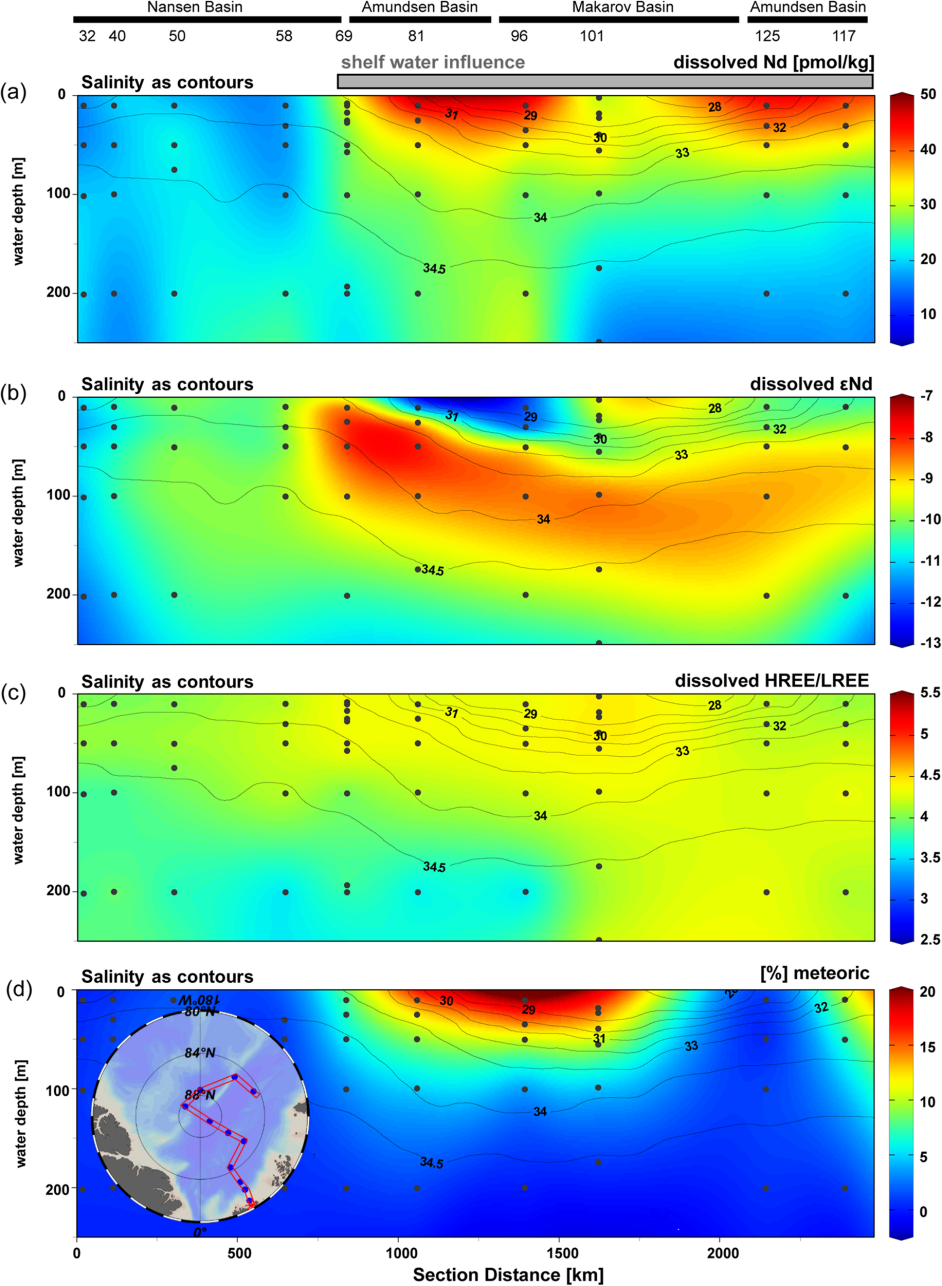
Distributions of REE concentrations, ε_Nd_ and meteoric fraction along the cruise track with salinity as contours. Transect from station 32 to 117 for the upper 200 m of dissolved (**a**) Nd concentrations, (**b**) ε_Nd_, (**c**) PAAS-normalized HREE/LREE ratios and (**d**) the fraction of meteoric water in color and salinity (Rabe et al., 2016^33^) as contours. Station numbers and basins are given on top of the transects, the transect is shown in the insert in panel (**d**). The figure was produced using Ocean Data View^64^ (version 5.1.7, https://odv.awi.de/) and modified manually.

The PAAS-normalized REE patterns of all samples mirror the typical characteristics of seawater with a pronounced Ce anomaly and an enrichment of heavy REEs (HREEs) over light REEs (LREEs) (Fig. S3), reflecting the stronger particle reactivity of LREEs compared to HREEs^34^. The HREE/LREE ratios along the transect, depicting scavenging-release behavior of the REEs, range between 3.1 and 4.7 (Fig. 3). Higher ratios (HREE/LREE = 4.0–4.7) that do not correlate with [Nd] are only observed in surface samples with meteoric water contribution (f_met_ > 2%, st. 69–125, Fig. S4). These high HREE/LREE ratios suggest REE scavenging in the Siberian river estuaries^26,35^ and no additional change along the TPD transport pathway. In the Lena estuary, such scavenging has been observed to cause higher LREE than HREE removal^26^, resulting in higher HREE/LREE ratios compared to those in the Atlantic water. Lower ratios (3.1–4.4) that correlate with [Nd] but not with [Er], occur in samples without meteoric contribution (stations 32–58 and samples from > 100 m depth) and support the presence of pristine Atlantic water as these HREE/LREE ratios are identical to those reported for Atlantic inflow from Fram Strait^6^. Enhanced [Nd] compared to the Atlantic inflow could be a result of some release of REEs from particles.

The distribution of dissolved ε_Nd_ along the transect supports our allocation of the stations with respect to TPD influence. Within the TPD, dissolved ε_Nd_ exhibits a wide range of values (ε_Nd_ = − 7.9 to − 12.2, Fig. 3b) and significant and strong vertical and lateral gradients for such a small depth and distance range in the open ocean, marking contributions from the Siberian rivers with their different characteristic ε_Nd_ signatures (see Introduction). In contrast, dissolved ε_Nd_ outside the TPD influence is in a narrow range (within ε_Nd_ = − 9.8 to − 11.8, getting more radiogenic along the flowpath of the waters), in line with a dominant Atlantic water influence (inflowing Atlantic water ε_Nd_ = − 11.7 at Fram Strait^6^) and no significant contributions from rivers or Pacific water (Fig. 2, Table S1). A pronounced feature within the TPD is a radiogenic Nd isotope band with ε_Nd_ = − 8.6 ± 0.2 (n = 5, st. 69–125) at 100 m water depth that shoals towards station 69 (Fig. 3b). These positive ε_Nd_ signatures suggest strong influence of a radiogenic ε_Nd_ source such as the Yenisei/Ob rivers or Pacific water.

### Dissolved REE and ε_Nd_ behavior in the central Arctic Ocean

The correlation of [REE] with the meteoric fraction and DOC (Fig. S2) in the TPD suggests predominant dissolved input of REEs via the Siberian rivers^11^, as DOC concentrations in Arctic rivers are high and mix conservatively in the estuaries^36^. Part of the scatter in the [REE]-meteoric water correlations may be linked to [REE] redistribution on the shelves due to sea-ice formation and melting^26^, which could also result in a decoupling of HREE/LREE ratios from the meteoric water fraction. During ice production, REEs can be incorporated into the ice, transported with the ice, and then released upon melting. These REE redistribution mechanisms are suggested to occur analogous to the redistribution of salt^37^. The scatter at higher meteoric water fractions is likely due to varying relative contributions of the Siberian rivers with their different [REE] and potential seasonal and interannual variations (Ob: 2152 pmol/kg Nd, early summer high discharge^27^, Yenisei: 154 pmol/kg Nd, early summer high discharge^27^; Lena: 477–824 pmol/kg Nd, early summer high discharge^24,26,27,38^; 744 pmol/kg Nd, winter discharge^26^).

The [Nd] in the TPD is further lower than discharge-weighted river contributions of [Nd]^11^ assuming 75% REE removal in the estuaries^26^, suggesting a Nd deficit in TPD surface waters and hence providing no direct evidence for REE contributions from particulate phases (either on the shelves or via Nd release from suspended particles). This is in line with observations from the Lena river and Severnaya Dvina river estuaries and Kara Sea freshwater, where the only documented process influencing dissolved [REE] is their removal in the low salinity region^26,35^. However, REE removal in the low salinity zones of the Lena and Dvina estuaries is not as strong as in other estuaries^26,35^, indicating fundamentally different riverine and estuarine settings that may also explain the lack of significant net release of REEs. On the one hand, the total load of suspended matter in the fluvial input to the Arctic Ocean is very low, about one order of magnitude lower than the global average river particle load^39^, which limits the potential for REE release. On the other hand, the composition of nanoparticles and colloids has been shown to influence the release behavior of REEs, with reduced REE release in rivers with a high ratio of organic to inorganic constituents^40^, such as in the Siberian rivers^36^, limiting REE release. The dominant process supplying REEs to the TPD is therefore dissolved riverine input, with little influence of REE release from particles or shelf sediments. This is in contrast with data from the Canada Basin^32^ and Chukchi Sea^25^, where shelf REE contributions were invoked. Furthermore, even if we cannot rule out any contribution of the Mackenzie river, it would be neglectable, given that most discharge of this river is distributed along the Canadian coast and seems to be restricted to 200–400 km into the Beaufort Sea^41,42^. High terrestrial dissolved input and conservative behavior in the TPD has previously been suggested for a number of other trace elements, DOC and silicic acid from previous cruises including the European (this study) and the US GEOTRACES cruises^2–8,11^. High terrestrial REE input is supported by the wide range of ε_Nd_ signatures in surface waters with high meteoric fractions and low salinities, that deviate from the dominant marine source of AW in this area.

Outside the TPD, where purely marine conditions with nearly 100% Atlantic fraction prevail even at the surface, the vertically constant [REE] distribution (Figs. 2, 3) is consistent with observations from the Canada Basin^32^ and the Fram Strait^6^. Yet, the [REE] distribution is unlike typical open ocean REE profiles, which exhibit surface REE depletions over deep water [REE] due to enhanced scavenging at the surface and REE release at depth^43–45^. This suggests little to no scavenging and export of REEs from the upper to the lower water column in the central Arctic Ocean. Evidence from Si isotopes and very low POC export fluxes in the Arctic indicate exceptionally low biogenic particle concentrations and a lack of significant biogenic particle dissolution in the deeper water column^46,47^. This can explain the uniquely constant REE profiles in areas and at depths not influenced by river input and highlights the important role of organic particles for the vertical redistribution of REEs^48^.

**Figure 4:**
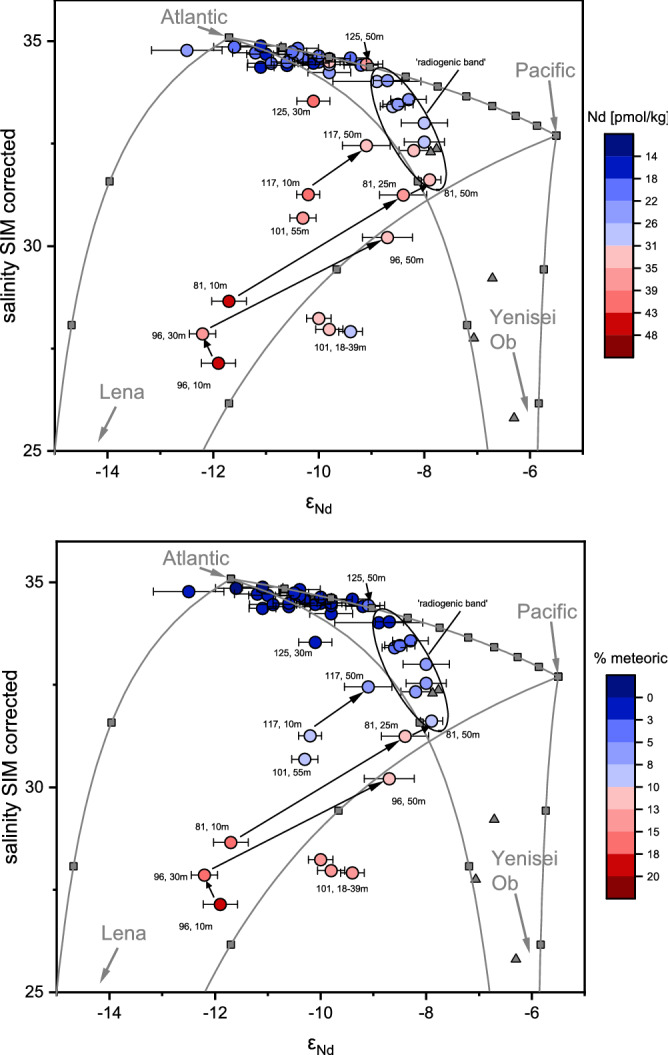
Mixing plots of ε_Nd_ vs. salinity. Data from samples from 10 to 200 m water depth with [Nd] in color (upper panel) and the meteoric fraction in color (lower panel). Error bars show the propagated errors (2SD, ε_Nd_). Grey lines represent mixing between the endmembers, grey squares show 10% mixing intervals, grey squares at the end of the mixing lines show the endmember Atlantic and Pacific. The black arrows mark the shift in ε_Nd_ from the very unradiogenic (Lena dominance) to more radiogenic (Yenisei/Ob dominance) values with water depths for selected stations. Grey triangles show samples from the Laptev Shelf close to Vilkitsky Strait^26^. The ‘radiogenic band’ comprises the samples at st. 69 10–100 m water depth, st. 81 50–100 m water depth and st. 96, 101 and 125 at 100 m water depth. Lena and Yenisei/Ob endmembers have a salinity of zero and thus are not shown but arrows point in their direction. Endmember values can be found in Table S2. The figure was produced using Origin (version 2019, https://www.originlab.com/) and modified manually.

Based on this evidence, we consider the dissolved [REE] and ε_Nd_ in the central Arctic Ocean upper water column to behave largely conservatively, and their signals in the TPD to be predominantly supplied as dissolved load by the Siberian rivers. Even if some particulate input from the riverbed sediments would occur, this would not change the ε_Nd_ signature significantly as the Siberian riverbed sediments show similar ε_Nd_ signatures as the dissolved fraction^26,49^. We therefore use dissolved [Nd] and ε_Nd_ together with salinity and the meteoric fraction estimates to distinguish between the different river contributions (Lena and Yenisei/Ob) within the TPD. The dissolved ε_Nd_ values along the cruise transect fall within the mixing envelopes of [Nd] versus ε_Nd_ (Fig. S1) and salinity versus ε_Nd_ (Fig. 4) defined by Pacific, Atlantic, Lena and Yenisei/Ob endmembers, further supporting largely conservative behavior of the measured [Nd] and ε_Nd_ along the transect. The large range of ε_Nd_ in the TPD reflects the different ε_Nd_ signatures of the potential endmembers of Atlantic water (ε_Nd_ = − 11.7^6^), modified Pacific water emerging from the Chukchi Shelf (ε_Nd_ = − 5.5^24,25^), and the rivers Lena (ε_Nd_ = − 15.7^26^), Yenisei (ε_Nd_ = − 5.2^27^), and Ob (ε_Nd_ = − 6.1^27^). Yenisei and Ob are combined to one endmember due to their similar Nd isotope signatures and discharge area in the Kara Sea resulting in a discharge-weighted combined ε_Nd_ signal of − 6.0.

A mathematical approach and attempt to quantify the relative contributions of all water masses mentioned above for the upper 500 m using salinity, oxygen isotopes, ε_Nd_ and [Nd] as an alternative for the water mass assessment based on salinity, oxygen isotopes and nutrient relationships is presented and discussed in the Supplementary Information. This approach is, however, hampered by the similarity of the ε_Nd_ signatures of Pacific and Yenisei/Ob waters and by the similarity of the ε_Nd_ signatures of Atlantic and Lena waters. Therefore, mixtures of Pacific and Lena, or of Atlantic and Yenisei/Ob can both result in identical salinity, ε_Nd_, and [Nd] values. In the northwestern Laptev Sea, for example, where Pacific water is inarguably not present, the Yenisei/Ob waters after mixing with AW and advection via the Vilkitsky Strait^50^ have characteristics^26^ indistinguishable from those of modified Pacific water emerging from the Chukchi Sea^24^. This demonstrates the unsuitability of this approach in quantifying the exact water component fractions for the entire upper water column of the central Arctic Ocean where waters from the Laptev and Chukchi seas could prevail. However, this method may prove useful if other parameters become available that allow differentiation of Pacific and Yenisei/Ob water in the Arctic Ocean. For example, a recent assessment of CDOM in the central Arctic Ocean^51^ revealed that different organic components can be distinguished by their fluorescence spectra, which could be helpful to tell Pacific and river water apart. For details see Supplementary Information.

Instead, we apply salinity and δ^18^O to determine sea-ice melt and meteoric fractions to assess the relative contributions of Lena and Yenisei/Ob to the TPD on the basis of Nd isotope signatures and [Nd]. We refrain from a quantitative assessment of the rivers Lena and Yenisei/Ob as this would depend on calculated (nutrient-based) Atlantic and Pacific fractions. The focus is on an independent qualitative and semi-quantitative assessment of the different freshwater and marine contributions to the TPD that yet provides crucial insight into the trace element sources and the TPD structure.

## Discussion

Least radiogenic Nd isotope signatures reaching ε_Nd_ = − 12.2, strongly elevated [Nd] and a high meteoric component at the surface of stations 81 and 96 suggest strongest Lena influence (Figs. 3b and 4), underlain by more radiogenic waters with ε_Nd_ around − 8 at 25 and 50 m depth, respectively. This pattern is also seen at stations 117 and 125, but with slightly more radiogenic surface ε_Nd_ of − 10.2 to − 10.5 and only a + 1 epsilon unit change towards underlying water/subsurface depths (Fig. 4). This distribution hints at a greater influence of Lena water at the very surface (down to maximum 30 m water depth) that is underlain by Yenisei/Ob water. At station 101 in the Makarov Basin, more radiogenic ε_Nd_ values (ε_Nd_ = − 9.7, average of the samples at 18–39 m water depth) are found at the surface (Figs. 3b, 4). At this station, input from the Kolyma river (ε_Nd_ = − 6.0^27^) that is discharged into the Canadian Basin (Makarov and Canada Basin), could be an alternative or additional radiogenic source to enhanced contribution of Yenisei/Ob and/or Pacific water.

The differences in the amount of Lena water between stations 81/96 and 117/125 can be explained by variations in Laptev Shelf hydrography: in September 2013, Laukert et al.^26^ found a prominent Lena signal in the central Laptev Sea that was then advected to the north and was incorporated in the TPD^26^. By September 2015, at the sampling time for this study, these waters could have reached stations 81 and 96 according to the speed of the TPD of 1–5 km/day^11,12^. In contrast, in September 2014, the Lena signal was weaker on the central Laptev Shelf (lower [Nd] and more radiogenic ε_Nd_ signal) and the Lena river water was more deflected to the east^26^, in agreement with a contrasting wind situation compared to 2013^52^. These shelf waters from 2014 could have reached stations 117 and 125 by September 2015. Therefore, the annual variability and different paths of the Lena river water found in 2013 and 2014 on the Laptev Shelf are consistent with the ε_Nd_ distribution in our dataset from the central Arctic Ocean in 2015.

The different depths of the Lena—Yenisei/Ob interface are likely the result of different density ranges of shelf waters entering the Arctic Ocean from the Kara and Laptev Seas^53^. The release of shelf waters across the frontal system along the continental shelf break occurs seasonally in pulses with large interannual variations^54^.Therefore, the different ε_Nd_ signals at our stations reflect different seasonal or annual contributions of river water which, together with the spatial fluctuations of the TPD, could account for the observed differences in the location and spatial extent of the Lena and Yenisei/Ob influence.

The radiogenic ε_Nd_ band with values of − 8.3 to − 8.9 at 100 m water depth (stations 69–125) that outcrops at station 69 (Figs. 3b, 4) suggests either enhanced Pacific and/or Yenisei/Ob contributions according to the mixing plot (Fig. 4). The radiogenic ε_Nd_ values are accompanied by elevated [Nd] of 24.3–35.9 pmol/kg and a salinity range of 31.7–34.4. In fact, the Nd concentrations in the radiogenic band are slightly lower compared to those in the Lena plume (on average 28 pmol/kg Nd in the radiogenic band compared to 42 pmol/kg Nd in the Lena dominated samples). Such concentrations, at only slightly elevated meteoric fractions and ε_Nd_ signatures around − 8, can also be found in the western Laptev Sea^26^ and the eastern Barents Sea^31^. Despite relatively low estimated meteoric fractions of 1–6% within this radiogenic band (Fig. 4), the river waters can still dominate the ε_Nd_ signal due to their very high [Nd] concentrations. Laukert et al.^31^, for example, clearly demonstrated for the eastern Barents Sea that a freshwater contribution of up to 2% Ob or 3–4% Ob/Yenisei to Atlantic Water (ε_Nd_ ~ − 12) would be sufficient to cause a shift in the ε_Nd_ signal to values around − 8, at Nd concentrations around 15 pmol/kg as a result of scavenging^31^.

Previous studies have suggested that Pacific water is restricted to the Canadian Basin with the Pacific front ranging from the Mendeleev Ridge to the Lomonosov Ridge and correlated with changes in atmospheric circulation^19,23,55–59^. Studies based on samples from 2015 show the front of Pacific water in the halocline at the Mendeleev Ridge^23^ or dominance of Pacific water up to the Lomonosov Ridge^60^ depending on the method. These estimates are based on transects in the Canadian Basin, therefore no information is available for the Eurasian side of the Arctic Ocean. Based on the following discussion we suggest that the radiogenic signal is rather caused by Yenisei/Ob water than Pacific water: even though Pacific water may have been present around the North Pole in 2015^23,60^, it is very unlikely that Pacific water is advected as far as station 69 in the Eurasian Basin in high amounts . Contributions of Pacific water calculated based on salinity, δ^18^O and N/P are highest at the surface at st. 96 and 101. Below the very surface and at the stations of the radiogenic band, the Pacific water fractions range between 4 and 20%. These Pacific values are considered maximum values, given that the N/P method ignores shelf processes altering the N to P towards lower N/P ratios and by doing so overestimates the Pacific contribution^3^. But these relatively low Pacific contributions in the radiogenic band cannot account for the corresponding ε_Nd_ signatures, as most of the samples would require a dominance of Pacific water to explain the very radiogenic signal. On the other hand, if there was a layer in the Eurasian Basin dominated by Pacific water, we would expect its ε_Nd_ signatures to approach − 6.4 to − 6.7, as seen in the Canadian Basin^24^. Furthermore, ε_Nd_, [REE], salinity and δ^18^O characteristics similar to those in the radiogenic ε_Nd_ band have been determined in the northwestern Laptev Sea^26^, where the freshwater component is dominated by Yenisei/Ob freshwater^26^ advected via the Vilkitsky Strait^50^, these samples are also shown in Fig. 4. This suggests that the northwestern Laptev Sea is the main source region of waters within the radiogenic ε_Nd_ band, supporting our hypothesis that this signal reflects the advection of Yenisei/Ob waters. We therefore conclude that Yenisei/Ob contribution and mixing with Atlantic water is mainly responsible for the consistently radiogenic ε_Nd_ band at stations 69–125. The outcropping of the signal at station 69 is probably a result of the absence of Lena river water overlying the band at the other stations, a setting that is also observed in the northwestern Laptev Sea where these waters likely originate from^26^.

The observations described above show that the river signals can still be distinguished far along the flow path of the TPD as the different river waters do not seem to mix entirely along the transport towards the Fram Strait. This contrasts with the idea of strong mixing of freshwaters from all Siberian rivers before advection to Fram Strait^6,37^, but is in line with limited mixing observed between Lena and Yenisei/Ob waters in the Laptev Sea^26^. The vertical and lateral separation of the river water from Lena and Yenisei/Ob can be explained by the river water flow paths before entering the TPD: water from the Yenisei and Ob originates in the Kara Sea, enters the open Arctic Ocean directly and/or flows through Vilkitsky Strait and then along/on the Laptev Shelf before entrainment into the TPD. Lena river water, on the other hand, enters from the Laptev Sea and is then directly incorporated into the TPD or deflected towards the east. Direct advection of Yenisei/Ob water through the Kara and Barents Seas to the Nansen and Amundsen Basins has not been observed^3^. Nevertheless, scavenging of river-borne REEs in the Siberian Shelf seas is slow, thus direct advection of Kara Sea freshwater to the TPD would result in concentrations reaching up to 50 pmol/kg Nd. Also, discharge-weighted mean Kara Sea freshwater [Nd] is 200 pmol/kg higher than Lena river summer [Nd]^26^. Therefore, our data suggest that direct input of Kara Sea water to the TPD must be rather limited given the lower [REE] (< 18 pmol/kg for Nd) determined in the western Barents Sea^31^ compared to the [REE] observed within the Yenisei/Ob-dominated parcel of the TPD (> 25 pmol/kg for Nd). Yenisei and Ob waters therefore have a longer transit time (some months) before entering the TPD, increasing the potential of mixing with seawater resulting in a higher salinity and density than Laptev Shelf waters containing Lena freshwater.

Due to the high [REE] of the river water compared to seawater, even after estuarine REE removal, the river endmember Nd isotope signatures are maintained upon mixing with seawater, whereas the salinity and density change is more apparent. This can, for example, be seen in the northwestern Laptev Sea where waters close to the Vilkitsky Strait with a salinity of 25.8–32.4 have a very positive Nd isotope signal of ε_Nd_ = − 6.3 to − 7.8, representing Yenisei/Ob influence, whereas samples at the edge of the northern Laptev Sea with salinities of 20.6–29.7 represent Lena freshwater influence with Nd isotope compositions ranging between ε_Nd_ = − 12.4 and − 15.6^26^. The shoaling of the positive ε_Nd_ signal towards station 69 is then due to the absence or reduced presence of Lena river water at the surface, leading to an outcropping of the Yenisei/Ob water.

Dissolved ε_Nd_ and [REE] provide clear insight into the lateral and vertical relative distribution of the different river waters, spatially and temporally varying input and transport of river water constituents into and across the central Arctic Ocean. The different river waters show a vertical and lateral separation with Lena water overriding Yenisei/Ob water throughout the transport route of the TPD. Due to their different densities acquired prior to incorporation into the TPD, there is little to no mixing between the freshwater contributions. The lateral separation is likely a result of temporally varying river discharge and changing wind patterns over the shelves differently affecting the river plumes. This knowledge of the contribution and distribution of the different rivers in the central Arctic Ocean may be especially valuable in the future, as e.g. potentially changing processes in the shelf areas may lead to changes in halocline properties. River input is expected to change in composition and volume^61^ due to the impact of climate warming especially on permafrost areas in the hinterland of the Siberian rivers. Notably, discharge of the Lena river, currently draining exclusively permanent permafrost hinterland^14^, may be expected to increase in response to climate-induced thawing of permafrost areas. With the direct incorporation of Lena water into the TPD and, as our results show, its dominant role in sustaining the salinity and density stratification in the central Arctic Ocean, this has important implications not only for the macro- and micronutrient and freshwater budgets, but also for the water column structure of the central Arctic Ocean and downstream in the North Atlantic. This in turn affects nutrient bioavailability and cycling, primary production and planktonic food webs downstream the TPD.

## Materials and methods

All seawater samples presented in this study were collected during R/V Polarstern cruise PS94 (ARKXXIX/3, GEOTRACES transect GN04, August to October 2015). Sample processing and spectrometric analysis of dissolved [REE] and ε_Nd_ in the laboratories of the Marine Isotope Geochemistry group at the ICBM of the University of Oldenburg followed established methods (see Supplementary Information). Intercalibration with other laboratories for quality control was achieved previously and through analysis of samples from a crossover station with another GEOTRACES cruise and replicate samples from a nearby station of a previous R/V Polarstern cruise (see Supplementary Information for full details). All [REE] and ε_Nd_ data are respectively reported with 1SD and 2SD uncertainties calculated based on repeat analyses of a seawater standard and certified reference material, respectively. All data are provided in the Supplementary Information.

## Supplementary Information

Supplementary Information.
