# Supplementary material for: Separating individual contributions of major Siberian rivers in the Transpolar Drift of the Arctic Ocean
Source: Sci Rep. 2021 Apr 15;11:8216. doi: 10.1038/s41598-021-86948-y (PMC8050230; doi:10.1038/s41598-021-86948-y)
Supplement: Supplementary file 1 — Supplementary Information. [file 41598_2021_86948_MOESM1_ESM.pdf]

Supplementary Information for

Separating individual contributions of major Siberian rivers in the Transpolar Drift of the Arctic Ocean

Ronja Paffrath[1*], Georgi Laukert[2], Dorothea Bauch[2,3], Michiel Rutgers van der Loeff[4], Katharina Pahnke[1]

[1] Marine Isotope Geochemistry, Institute for Chemistry and Biology of the Marine Environment (ICBM), University of Oldenburg, Carl-von-Ossietzky-Str. 9-11, 26129 Oldenburg, Germany

[2] GEOMAR Helmholtz Centre for Ocean Research Kiel, Wischhofstr. 1-3, 24148 Kiel, Germany

[3] Leibniz-Labor, University of Kiel (CAU), Max-Eyth-Str. 11-13, 24118 Kiel, Germany

[4] Alfred Wegener Institute, Helmholtz Centre for Polar and Marine Research, Am Handelshafen 12, 27570 Bremerhaven, Germany

* Correspondence and requests for materials should be addressed to R.P.
(email: ronja.paffrath@uni-oldenburg.de)

Methods

Sampling and onboard procedures

Seawater samples were collected at 10 stations during R/V Polarstern cruise PS94 (ARKXXIX/3, GEOTRACES transect GN04, August to October 2015) from Tromsø, Norway, to Bremerhaven, Germany. Seawater was sampled from 24L-Niskin bottles by following GEOTRACES protocols (GEOTRACES Cookbook[1]). The water was directly filtered from the Niskin bottles through AcroPak^{TM}500 Capsules with Supor Membrane (pore size: 0.8/0.2 µm) into acid-cleaned LDPE containers for Nd isotope analyses and into HDPE bottles for REE concentration analyses using Teflon-lined Tygon tubing. The filter cartridges were precleaned with seawater prior to sample collection and rinsed with MQ water between the stations. The cartridges were used for approximately the same depths at each station. Samples for Nd isotopes were preconcentrated using one SepPak C18 cartridge (Waters Inc.) per 5 or 10 L and were preloaded with 300 mg of 2-ethylhexyl phosphate (HDEHP, Merck) using a method modified after Shabani et al.[2] and Jeandel et al.[3]. Samples for [REE] were acidified to a pH < 2 using 6 N ultrapure distilled HCl (Teflon still). MQ water from the onboard system used to rinse the filters was collected to obtain total procedural blanks.

Neodymium isotope preconcentration and analysis

In the home laboratory, every C18 cartridge was rinsed with 5 mL 0.01 N ultrapure distilled HCl to remove remaining Ba and the REEs were eluted using 35 mL ultrapure distilled 6 N HCl following established methods[2,3]. Nd was purified using two-step chromatography[4]. The first column filled with Triskem TRU resin (particle size 100– 150 µm) was used to remove remaining HDEHP from the cartridge. In a second step, the Nd was separated from the other REEs using Triskem LN resin (particle size 100– 150 µm). HCl and HNO_3 used for these procedures were all ultraclean distilled.

The Nd isotopes ($^{143}Nd/^{144}Nd$, expressed as $\varepsilon_{Nd} = [[(^{143}Nd/^{144}Nd)_{sample}/(^{143}Nd/^{144}Nd)_{CHUR}] - 1] \times 10^4$, with CHUR: Chondritic Uniform reservoir[5]) were analyzed using a Thermo Scientific *Neptune Plus* multi-collector inductively coupled plasma mass spectrometer (MC-ICP-MS) at the ICBM in Oldenburg. A Cetac *Aridus II* desolvating nebulizer system was used for sample introduction. The samples were

measured for 1 x 36 cycles at concentrations ranging between 5 and 20 ppb Nd in 250 µL solution with peak centering prior to the measurement session. The Nd standard JNdi-1 was measured after every 2-3 samples at the same concentration as the samples. The measured $^{143}Nd/^{144}Nd$ ratios were corrected for the instrumental mass fractionation using an exponential law and $^{146}Nd/^{144}Nd = 0.7219$[6]. If possible (positive correlation between $^{143}Nd/^{144}Nd$ and $^{142}Nd/^{144}Nd$), we applied a secondary mass bias correction using a linear correlation[7] of $^{143}Nd/^{144}Nd$ and $^{142}Nd/^{144}Nd$. All data were normalized to the accepted value for the JNdi-1 standard[8] of $^{143}Nd/^{144}Nd = 0.512115$. The external reproducibility was monitored with repeated measurements of JNdi-1 and an in-house Nd standard, with Nd concentrations matching those of the samples, during the measurement sessions and for JNdi-1 was typically better than ± 0.4 ε_{Nd} units (2SD, n = 4–16 per session). The analysis of one duplicate sample and repeated measurements of 6 samples showed agreement within the analytical uncertainty. In Table S1, the internal, external and propagated errors (calculated from internal and external error, 2SD) are shown. Blanks were processed identically to the samples and spiked with a ^{146}Nd spike for Nd quantification via MC-ICP-MS. Total procedural blanks (n = 3) from the shipboard MQ system were < 37 pg Nd, the procedural laboratory blanks (n = 16) were < 6 pg Nd, which represent < 3 % and < 1 % of the lowest sample concentration, respectively.

Rare earth element preconcentration and analysis

For the determination of [REE], isotope dilution ICP-MS analysis was applied following the method described in Behrens et al.[9]. Briefly, 10-20 mL seawater aliquots were spiked with a multi-element REE spike, processed with an automated seaFAST-pico system (ESI) in offline mode for REE preconcentration and matrix removal, and REEs were analyzed using a Thermo Finnigan *Element II* ICP-MS in combination with a Cetac *Aridus II* desolvating nebulizer system. Nitrogen supply allowed for reduction of oxide formation to < 0.04 % for Ce-oxide, corrections for oxide formation were therefore not necessary. For accuracy and external reproducibility, the seawater standard SAFe 3000 m (n = 13) was used. The values agreed well within 7 % of the published average [REE] of four different labs[9] and showed a reproducibility of < 10 % (2SD) and 35 % for Ce. Total procedural blanks from shipboard MQ water, procedural lab blanks (2 % HNO_3, distilled, seaFAST preconcentration), and analytical blanks (2 % HNO_3, distilled) were in general < 1.8 % (< 10 % for Ce) of the lowest

sample concentration. The blanks were spiked for isotope dilution analysis after preconcentration and/or prior to the measurement. The ratios of HREE/LREE were calculated after Martin et al.[10] after REE normalization to Post Archean Australian Shale (PAAS[11]). The standard deviation of the standard SAFe 3000 m (n = 12) for HREE/LREE was 4.6 % (2SD).

Intercalibration

The laboratory at the ICBM in Oldenburg is intercalibrated for [REE] through analysis of GEOTRACES standard SAFe 3000 m[9] and for Nd isotopes through analysis of GEOTRACES intercalibration samples BATS 15 m and 2000 m and SAFe 3000 m[12]. Comparison of REE analyses from a crossover station in the central Arctic Ocean (station PS94-101) of this cruise and R/V Healy cruise HLY1502 (station 30; analyzed by Brian Haley, CEOAS, Oregon State University) show agreement within 4.6 % for Nd and 9.3 % for Er (water depth ≥ 1000m[13]). Neodymium isotope analyses of two replicate samples from the Fram Strait (sample ID: PS100, station 125, 10 m and 150 m) measured at GEOMAR and at ICBM (G. Laukert and R. Paffrath, unpubl. data) show agreement within the analytical uncertainty.

Calculation of water mass fractions based on salinity, $\delta^{18}O$ and N/P ratios

Salinity and stable oxygen isotopes ($\delta^{18}O$) combined have been applied to determine the fractions of a marine component, meteoric water and sea-ice melt (SIM)[14]. The $\delta^{18}O$ composition of the water is a conservative property that is altered by fractionation due to phase transitions only. Uncertainties in water mass fractions in the 3-component water mass analysis based on salinity and $\delta^{18}O$ therefore depend on errors of the chosen endmembers only and result in median standard deviations of 1% and 0.6% for meteoric water and sea-ice meltwater, respectively[15]. The 3-component water mass analysis was complemented by nutrient relationships (e.g. N/P and/or PO_4*) in the early 2000s to allow for a 4-component mass-balance enabling further discrimination of the marine component into Atlantic and Pacific water[16–19]. This approach, however, remains challenging due to shelf processes producing nutrient signatures in Siberian shelf derived waters similar to Pacific waters[19,20] and differences in estimates of Pacific water fractions based on the different methods are of up to 60 and 40 % in the central Arctic Ocean and Fram Strait, respectively[15,21]. Therefore,

we refrain here from using Atlantic and Pacific fractions from the 4-component mass-balance but use the meteoric water fraction and SIM-corrected salinities, which are identical within error with those determined based on the 3-component analysis. The few values referred to for fractions of Pacific water based on the N/P method were calculated as described by Bauch et al.[19].

Calculation of water mass fractions based on salinity, $\delta^{18}O$, [Nd], ε_{Nd} and mass conservation (Nd-method)

Calculation of water masses is performed using a two-step method solving linear equations: In a first step, the fractions of marine water (mar), meteoric water (met) and sea-ice melt (SIM) are calculated using salinity and $\delta^{18}O$:

$$1 = f_{mar} + f_{met} + f_{SIM} \quad (1)$$

$$S_{meas} = f_{mar} * S_{mar} + f_{met} * S_{met} + f_{SIM} * S_{SIM} \quad (2)$$

$$\delta^{18}O_{meas} = f_{mar} * \delta^{18}O_{mar} + f_{met} * \delta^{18}O_{met} + f_{SIM} * \delta^{18}O_{SIM} \quad (3)$$

For water depths without $\delta^{18}O$ measurements, the $\delta^{18}O$ value was interpolated from over- and underlying samples (Supplementary Table 2). Calculated meteoric fractions are used for further qualitative evaluation (presented in Figs. 3+4 of main article) but are not further used for Nd-based calculations. Sea-ice melt/formation cannot be directly considered in the water mass calculations using [Nd] and ε_{Nd} as the effect of sea-ice melt/formation on these parameters is not well understood. It was suggested before that incorporation of Nd into sea ice is proportional to salinity changes and that ε_{Nd} of the formation region is preserved in the sea ice[22]. However, REEs are only incorporated into the sea ice[23] to a small extent, therefore changes in [Nd] or ε_{Nd} related to SIM are not considered directly in our study as the SIM is considered to be relatively low (< 8 %). Nonetheless, the salinity is corrected for the fraction of SIM as described in Rósen et al. (2015)[24].

The salinity corrected for SIM together with measured [Nd] and ε_{Nd} is used to calculate the water mass fractions. Endmembers used are Atlantic, Pacific, Lena and combined Yenisei/Ob river water, given their very similar [Nd] and ε_{Nd} compositions. The endmember values are listed in Supplementary Table S2.

$$1 = f_A + f_P + f_L + f_{Y/O} \quad (4)$$

$$S_{SIM\text{-}corrected} = f_A * S_A + f_P * S_P + f_L * S_L + f_{Y/O} * S_{Y/O} \quad (5)$$

$$[Nd]_{meas} = f_A * [Nd]_A + f_P * [Nd]_P + f_L * [Nd]_L + f_{Y/O} * [Nd]_{Y/O} \quad (6)$$

$$\varepsilon Nd_{meas} = \frac{f_A * \varepsilon Nd_A * [Nd]_A + f_P * \varepsilon Nd_P * [Nd]_P + f_L * \varepsilon Nd_L * [Nd]_L + f_{Y/O} * \varepsilon Nd_{Y/O} * [Nd]_{Y/O}}{f_A * [Nd]_A + f_P * [Nd]_P + f_L * [Nd]_L + f_{Y/O} * [Nd]_{Y/O}} \quad (7)$$

To obtain a set of linear equations, equations (6) and (7) can be replaced by equations (8) and (9):

$$[^{143}Nd]_{meas} = f_A * [^{143}Nd]_A + f_P * [^{143}Nd]_P + f_L * [^{143}Nd]_L + f_{Y/O} * [^{143}Nd]_{Y/O} \quad (8)$$

$$[^{144}Nd]_{meas} = f_A * [^{144}Nd]_A + f_P * [^{144}Nd]_P + f_L * [^{144}Nd]_L + f_{Y/O} * [^{144}Nd]_{Y/O} \quad (9)$$

with

$$[^{144}Nd]_{meas} = 0.238 * [Nd]_{meas}$$

$$[^{143}Nd]_{meas} = \left(\frac{\varepsilon Nd_{meas}}{10{,}000} + 1\right) * \frac{^{143}Nd}{^{144}Nd}_{CHUR} * [^{144}Nd]_{meas}$$

where f_A, f_P, f_L and $f_{Y/O}$ refer to the fractions of Atlantic, Pacific, Lena and Yenisei/Ob, respectively, and S, [Nd] and ε_{Nd} with subscripts are endmember values: SIM-corrected salinity and measured values of Nd concentration and ε_{Nd}. Differences between measured and calculated (based on the water mass fractions) values for all parameters are a measure for the quality of the calculations for the water masses and are comparably low as for the method based on oxygen isotopes and nutrients.

Discussion

Limitations of the water mass inventory based on the Nd-method

Applying the Nd-method with the four endmembers Atlantic, Pacific, Lena and Yenisei/Ob in the central Arctic Ocean, more than one combination of the four endmembers is possible to describe the parameters adequately for some of our samples. This is due to the insufficiently distinct endmembers of the chosen parameters. In fact, slight variations of the parameters within analytical error can lead to considerably different solutions for the calculated water mass fractions. The main problem arises from the similar ε_{Nd} signatures of

Pacific and Yenisei/Ob and of Atlantic and Lena waters, so that mixtures of e.g. Pacific and Lena or Atlantic and Yenisei/Ob can both result in identical salinity, ε_{Nd}, and [Nd] values. That is, for some samples there is more than one possible solution to mix the endmembers adequately to obtain the measured values for the respective parameters.

Further limitations are the endmember compositions: the river endmembers for Yenisei and Ob were only measured once and there is no information available about the seasonal or inter-annual differences in the Nd supply to the open ocean. Additionally, the calculations account for 75 % Nd removal but the actual removal may be lower in waters advected to the central Arctic Ocean in less time than it takes to reach maximal removal (e.g. possibly in years with fast and direct northward advection of the Lena plume). To a smaller extent, additional endmembers like the Kolyma river could contribute to the central Arctic Ocean that are not considered in the 4-endmember calculation. Seawater-sediment interactions may also result in non-conservative changes in ε_{Nd} and [Nd], as for example suggested for the Chukchi Shelf[25]. Such interactions could, however, not be confirmed for the Laptev and Barents Sea shelves[23,26] and instead sea-ice processes were suggested to influence [Nd] on the shallow Arctic shelves[23]. Currently the impact of sea-ice formation and melting on results of the Nd-method cannot be assessed given the unknown element fluxes during these processes and given the strong variability in the initial seawater concentrations, but these processes do not appear to significantly decouple [Nd] and ε_{Nd} distributions on the basin scale.

Even though Nd isotopes have been shown to be water mass tracers and have been used for this purpose in various studies[27–29], this quantitative Nd-method cannot be unambiguously applied to give a unique solution in the central Arctic Ocean. However, even though the method is not applicable in the setting presented here and with the available parameters, it may be a suitable complement to the methods based on oxygen isotopes and nutrient relationships in other regions where the endmember parameters used are more distinct. For the central Arctic Ocean, it may be useful if other parameters become available for the differentiation of Pacific and Yenisei/Ob water (to complement the Nd-method) or Atlantic and Pacific water (as a replacement for nutrient relationships). For example, recent assessment of CDOM (colored dissolved organic matter) in the central Arctic Ocean[30] shows that different organic components can be distinguished by their fluorescence spectra, which could be helpful to tell Pacific and river water apart.

Supplementary Figures

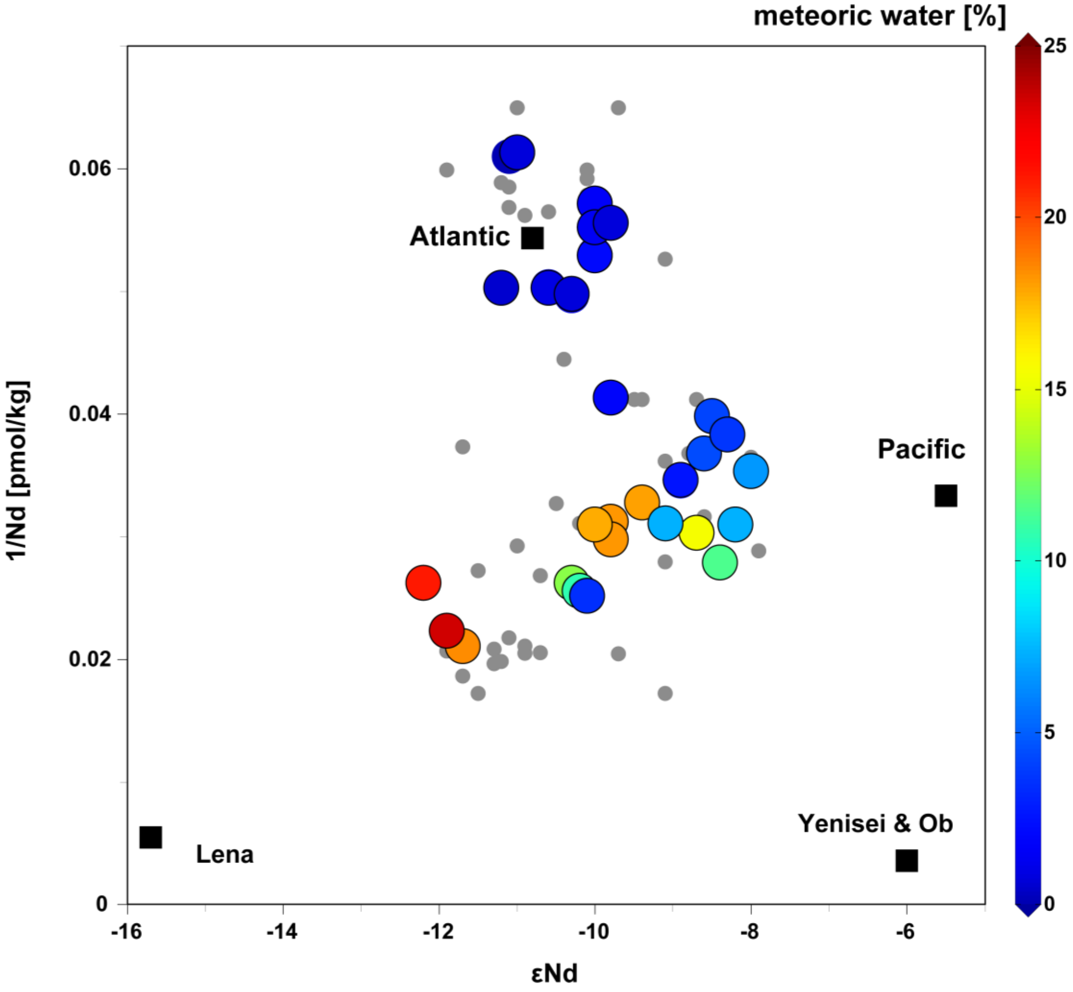

Figure S1. ε_{Nd} *vs.* 1/[Nd] of samples ≤ 100 m water depth. Endmembers are presented as black squares. The meteoric fraction is presented in color. Small grey dots represent samples from the Central Arctic from other studies (Andersson et al., 2008; Porcelli et al, 2009; Zimmermann et al., 2009). The figure was produced using Ocean Data View (Schlitzer, 2018) and modified manually.

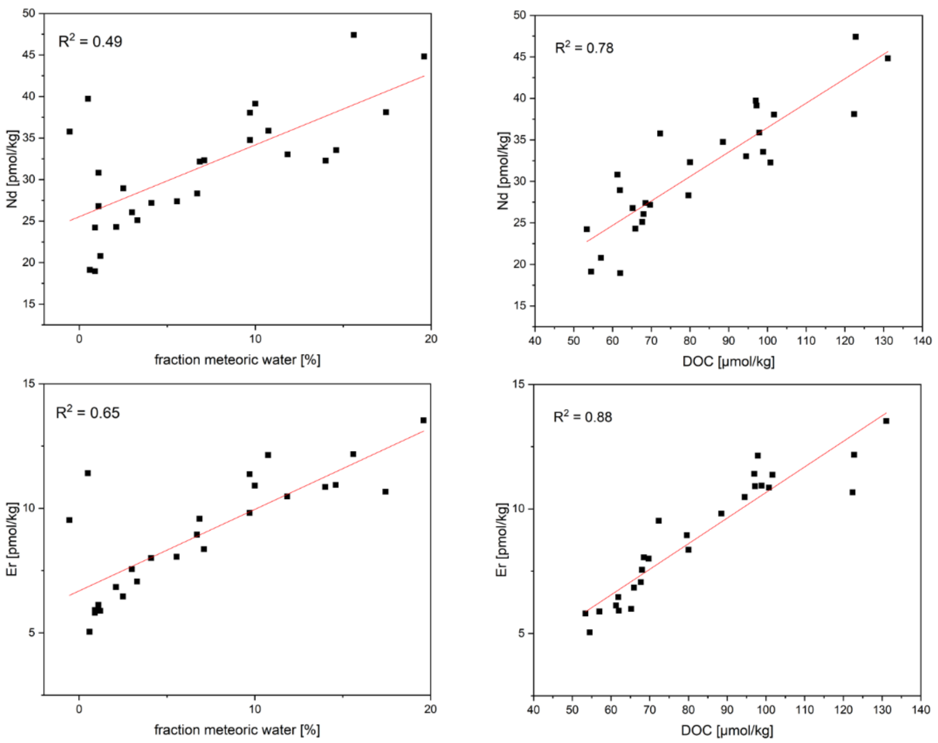

Figure S2. Relationship between REE concentrations and indicators of river water. Correlation of Nd concentration (upper panels) and Er concentration (lower panel) with the fraction of meteoric water (left; calculated via oxygen isotopes and salinity) and DOC (right; Charette et al.[13]) for samples ≤ 200 m water depth in the TPD (st. 69-125). P-values for all correlations are < 0.05.

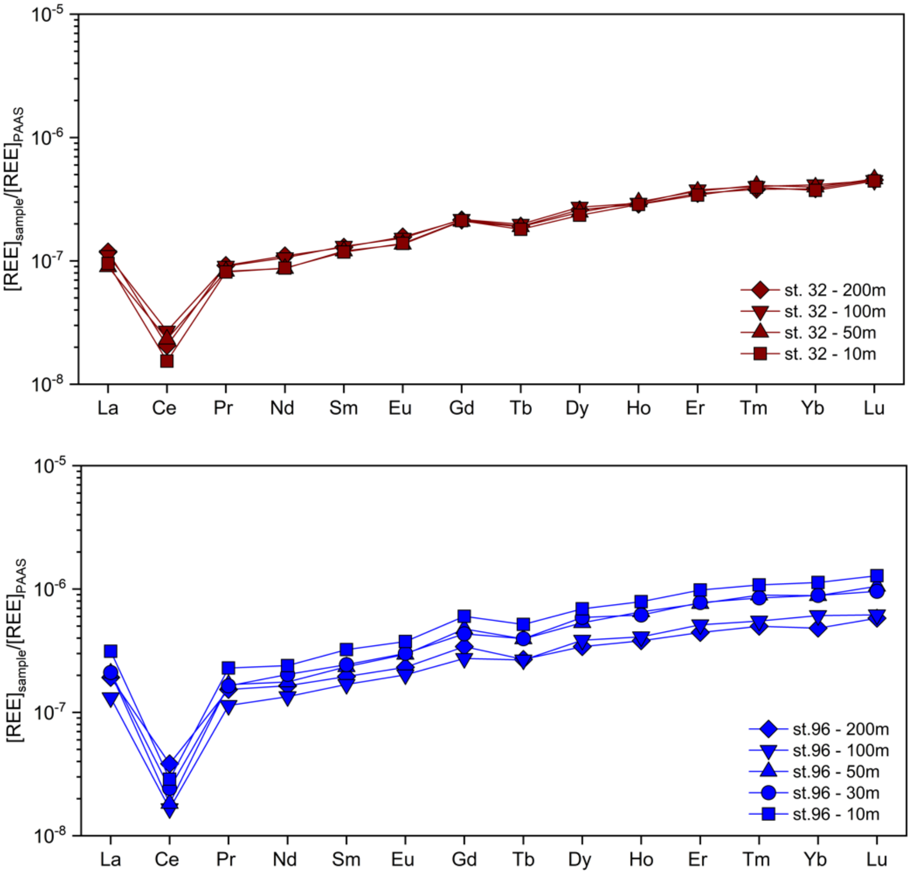

Figure S3. Examples of REE patterns of an Atlantic-only influenced station (32) and a river and Pacific influenced station (96).

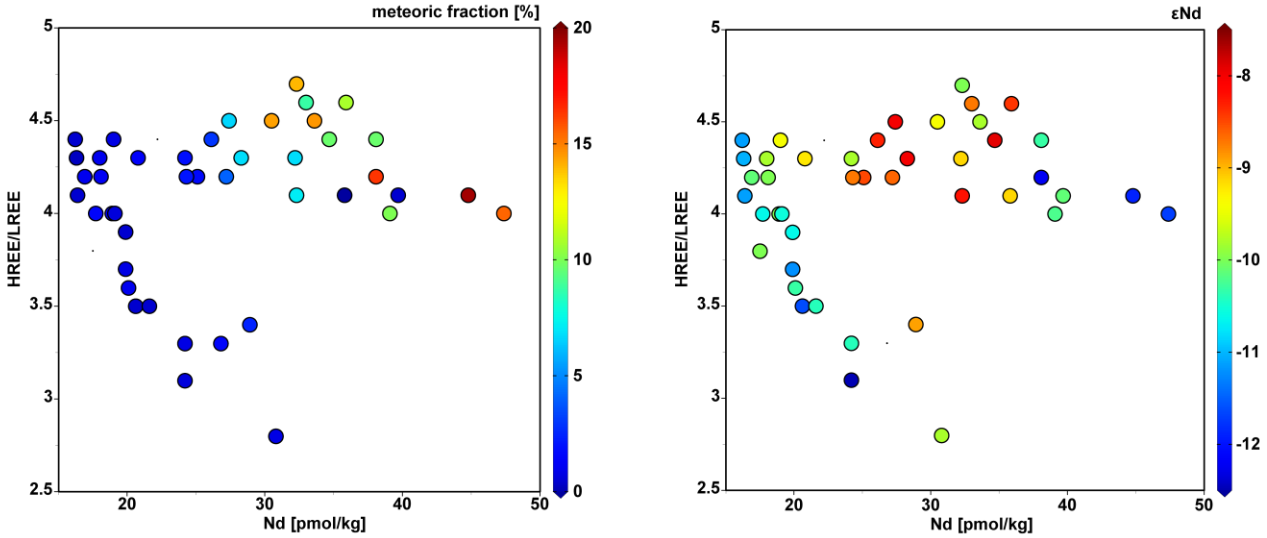

Figure S4. Neodymium concentrations *vs.* PAAS-normalized HREE/LREE with the meteoric fraction [%] (left panel) and the ε_{Nd} signature (right panel) in color for the top 200 m. The figure was produced using Ocean Data View[31] and modified manually. The errors for [Nd] and HREE/LREE ratios are 1.9 % and 2.3 % (1RSD), respectively.

Table S1. Hydrographic data[32], DOC[13], oxygen isotopes, REE and Nd isotopes. Note that the hydrographic parameters are bottle data for REE and Nd isotope samples, DOC and oxygen isotope samples are partly sampled from different bottles/casts. Values in italics are calculated from samples from other (above and below) water depths.

Station	Cast	Bottle	Latitude	Longitude	Depth	Temperature	Salinity	$\delta^{18}O$	sea-ice melt (SIM)	meteoric water	SIM-corr Salinity	DOC	La	Ce	Pr	Nd	Sm	Eu	Gd	Tb	Dy	Ho	Er	Tm	Yb	Lu	HREE/LREE	ε_{Nd}	int. error	ext. error	prop. error	sec. corr.
		[#]	[°N]	[°E]	[m]	[°C]		[‰]	[%]	[%]		[µmol/kg]							[pmol/kg]													
PS94-32	9	18	81.8597	30.8387	10.4	-1.30	32.784	0.103	5.4	0.5	34.367	60.8	21.2	6.9	4.1	16.4	3.7	0.9	5.4	0.8	5.6	1.4	4.7	0.7	4.3	0.8	4.1	-11.1	0.2	0.2	0.3	x
PS94-32	9	16	81.8597	30.8387	50	-1.12	34.323	*0.141*	*1.5*	0.2	34.686		20.0	10.4	4.2	16.3	3.8	0.9	5.3	0.8	6.0	1.5	5.1	0.7	4.6	0.8	4.3	-11.0	0.3	0.2	0.4	x
PS94-32	9	13	81.8597	30.8387	101.1	1.69	34.781	0.182	-0.3	0.9	34.720	56.1	24.7	12.1	4.6	19.9	4.1	1.0	5.5	0.9	6.6	1.5	5.2	0.7	4.8	0.8	3.7	-11.2	0.3	0.3	0.4	x
PS94-32	9	10	81.8597	30.8387	201.3	2.66	34.985	0.262	-0.5	0.5	34.861	59.5	26.5	9.3	4.6	20.6	4.0	1.0	5.5	0.8	6.2	1.5	4.9	0.7	4.4	0.8	3.5	-11.6	0.3	0.2	0.4	x
PS94-40	3	23	82.7065	30.9177	9.6	-1.52	33.633	0.085	3.4	0.4	34.492	58.3	25.1	11.6	4.8	19.9	3.9	1.1	5.6	0.9	6.5	1.6	5.2	0.8	5.0	0.8	3.9	-10.6	0.2	0.2	0.3	x
PS94-40	3	19	82.7065	30.9177	29.8	-1.70	34.294	*0.013*	*0.7*	1.3	34.460																	-10.9	0.3	0.3	0.4	x
PS94-40	3	16	82.7065	30.9177	50	-1.72	34.395	*-0.004*	*0.1*	1.5	34.410		22.0	11.0	4.2	17.7	3.5	0.9	5.4	0.8	5.9	1.4	4.9	0.7	4.4	0.8	4.0	-10.6	0.3	0.3	0.4	x
PS94-40	3	9	82.7065	30.9177	100	-0.41	34.562	0.113	0.1	1.0	34.593	59.7	25.4	14.1	5.4	20.1	4.6	1.1	6.0	1.0	6.3	1.7	5.2	0.8	4.9	0.8	3.6	-10.3	0.2	0.2	0.3	x
PS94-40	3	7	82.7065	30.9177	199.9	2.30	34.946	0.285	-0.2	0.4	34.884	56.5	19.6	7.3	4.0	16.2	3.4	0.9	4.7	0.8	6.1	1.4	5.1	0.7	4.8	0.8	4.4	-11.1	0.3	0.2	0.4	x
PS94-50	8	20	84.3987	30.7152	10.3	-1.52	33.636	-0.114	3.3	1.7	34.495	54.9	21.7	10.3	4.3	18.9	3.7	1.0	5.6	0.8	6.2	1.4	5.0	0.7	4.6	0.8	4.0	-10.0	0.3	0.3	0.4	x
PS94-50	8	10	84.3987	30.7152	50.1	-1.78	34.449	*-0.104*	*-0.7*	2.0	34.236		28.8	10.9	5.9	24.2	5.3	1.3	7.9	1.2	8.5	2.2	7.1	1.1	6.5	1.1	4.3	-9.8	0.3	0.3	0.4	x
PS94-50	8	2	84.3987	30.7152	200.4	-1.75	34.896	0.239	-0.2	0.5	34.827	53.7	27.0	11.5	5.1	21.6	4.4	1.1	5.8	0.9	7.0	1.5	5.5	0.7	4.8	0.8	3.5	-10.4	0.3	0.2	0.4	x
PS94-58	5	21	85.2798	60.0495	9.9	-1.76	33.252	-0.040	3.4	1.7	34.246	55.9	23.3	12.4	4.0	17.5	3.3	0.9	4.6	0.7	6.1	1.3	4.7	0.6	4.4	0.7	3.8	-10.0	0.2	0.2	0.2	x
PS94-58	5	11	85.2798	60.0495	30.2	-1.59	34.235	*0.018*	*1.7*	1.1	34.644		21.8	8.0	4.2	18.1	3.7	1.0	5.5	0.9	6.1	1.5	5.0	0.7	4.8	0.8	4.2	-10.0	0.1	0.2	0.2	x
PS94-58	5	6	85.2798	60.0495	49.8	-1.74	34.436	*0.031*	*0.8*	1.0	34.466		20.3	10.6	4.7	16.9	4.1	1.0	5.5	0.9	6.3	1.6	5.2	0.8	5.1	0.8	4.2	-10.1	0.3	0.1	0.3	x
PS94-58	3	21	85.2798	60.0495	100.3	-0.41	34.576	0.108	0.0	1.0	34.576	53.9	21.3	9.7	4.3	18.0	3.8	1.0	5.1	0.8	6.8	1.5	5.6	0.7	5.2	0.8	4.3	-9.8	0.2	0.3	0.4	x
PS94-58	3	10	85.2798	60.0495	200.4	1.63	34.871	0.209	-0.4	0.7	34.778		30.9	14.0	5.6	24.2	4.7	1.2	5.9	0.9	6.7	1.5	5.2	0.7	4.8	0.8	3.1	-12.5	0.5	0.4	0.7	
PS94-69	5	19	86.9982	58.7307	10.2	-1.65	33.118	-1.169	-3.0	7.1	32.332	80.0	37.8	13.8	6.8	32.3	5.9	1.8	9.0	1.4	10.9	2.0	8.4	1.1	8.3	1.4	4.1	-8.2	0.2	0.2	0.3	x
PS94-69	5	15	86.9982	58.7307	25	-1.67	33.707	*-0.841*	*-4.5*	6.8	32.539		34.2	11.6	7.3	28.3	6.5	1.7	9.1	1.5	11.0	2.3	8.9	1.1	8.5	1.4	4.3	-8.0	0.2	0.2	0.3	x
PS94-69	5	8	86.9982	58.7307	50	-1.78	34.011	*-0.225*	*-4.5*	6.7	33.006		30.1	12.7	7.0	27.4	6.1	1.8	8.6	1.5	10.0	2.5	8.1	1.2	8.0	1.3	4.5	-8.0	0.3	0.2	0.4	x
PS94-69	5	2	86.9982	58.7307	100.4	-0.88	34.355	0.160	-1.2	2.5	34.021	61.9	35.8	16.9	6.7	28.9	5.7	1.5	7.8	1.2	8.6	2.0	6.5	1.0	6.2	1.0	3.4	-8.9	0.3	0.3	0.4	x
PS94-69	4	20	86.9982	58.7307	200.2	1.11	34.829	0.193	-0.6	0.9	34.675	53.4	30.7	15.8	6.2	24.2	5.2	1.2	6.5	1.0	7.4	1.7	5.8	0.8	5.3	0.9	3.3	-10.4	0.8	0.3	0.8	x
PS94-81	7	14	88.9895	61.0633	10.3	-1.46	30.283	-3.382	-6.0	15.6	28.653	122.8	64.9	13.8	10.5	47.4	9.6	2.5	14.7	2.1	15.7	3.9	12.2	1.8	12.3	2.2	4.0	-11.7	0.2	0.2	0.3	x
PS94-81	7	10	88.9895	61.0633	25.2	-1.60	32.690	*-2.227*	*-6.1*	10.8	31.245		42.5	13.8	9.2	35.9	8.7	2.1	11.6	1.9	14.0	3.4	12.1	1.7	11.5	1.9	4.6	-8.4	0.4	0.2	0.4	x
PS94-81	7	5	88.9895	61.0633	50	-1.78	33.366	*-1.694*	*-6.9*	9.7	31.619		41.9	12.2	7.5	34.7	6.9	1.9	10.8	1.6	12.1	3.0	9.8	1.4	9.5	1.7	4.4	-7.9	0.2	0.1	0.2	x
PS94-81	5	24	88.9895	61.0633	99.6	-1.54	34.070	-0.592	-2.5	4.1	33.408	69.7	32.0	11.5	6.9	27.2	6.1	1.5	8.4	1.4	9.7	2.3	8.0	1.2	7.6	1.3	4.2	-8.6	0.2	0.2	0.2	x
PS94-81	5	17	88.9895	61.0633	174.4	0.08	34.585	*-0.015*	-0.8	1.5	34.432		32.0	14.4	6.0	26.8	5.1	1.3	6.8	1.0	7.9	1.7	6.0	0.8	5.5	0.9	3.3	-9.8	0.4	0.3	0.5	
PS94-96	7	23	88.3598	-125.09	10.2	-1.52	28.365	*-3.693*	*-5.3*	19.5	27.147	131.1	69.7	12.9	11.5	44.8	10.1	2.5	15.3	2.3	16.6	4.0	13.5	1.9	13.1	2.3	4.1	-11.9	0.2	0.1	0.2	x
PS94-96	7	16	88.3598	-125.09	30.6	-1.53	29.528	*-2.855*	*-7.0*	16.4	27.860		47.0	10.9	8.3	38.1	7.6	2.0	11.0	1.7	14.1	3.1	10.7	1.5	10.3	1.7	4.2	-12.2	0.2	0.2	0.3	x
PS94-96	7	14	88.3598	-125.09	50.9	-1.60	31.736	*-0.587*	*-6.4*	8.8	30.211		46.4	8.2	8.5	33.0	7.3	1.9	12.1	1.7	12.8	3.3	10.5	1.6	10.2	1.9	4.6	-8.7	0.1	0.2	0.2	
PS94-96	7	10	88.3598	-125.09	101.2	-1.42	33.971	0.061	-1.8	1.8	33.461	67.7	29.3	7.4	5.7	25.1	5.3	1.3	7.0	1.2	9.2	2.1	7.1	1.0	7.0	1.1	4.2	-8.5	0.4	0.2	0.5	x
PS94-96	7	4	88.3598	-125.09	202.3	0.20	34.623	0.125	-0.4	0.9	34.501	61.3	42.9	17.2	7.7	30.8	6.1	1.5	8.7	1.2	8.2	1.9	6.1	0.9	5.6	1.0	2.8	-9.8	0.2	0.2	0.3	x
PS94-101	9	19	87.4973	179.841	18.2	-1.50	28.132	*-3.779*	*-1.0*	14.5	27.915		43.6	7.5	7.1	30.5	6.6	1.8	11.4	1.6	11.4	2.9	9.3	1.4	9.0	1.7	4.5	-9.4	0.2	0.2	0.2	x
PS94-101	9	15	87.4973	179.841	22.6	-1.41	28.685	-3.650	-2.5	14.6	27.969	98.9	44.6	8.0	7.5	33.6	7.2	1.9	11.6	1.7	13.3	3.1	10.9	1.5	10.0	1.7	4.5	-9.8	0.2	0.2	0.2	x
PS94-101	9	10	87.4973	179.841	39.2	-1.44	29.010	-2.224	-2.7	14.0	28.235	100.8	42.9	7.7	7.8	32.3	7.4	1.9	11.8	1.8	13.2	3.2	10.9	1.5	10.3	1.8	4.7	-10.0	0.2	0.2	0.3	x
PS94-101	9	4	87.4973	179.841	55.4	-1.49	32.122	-0.483	-4.7	9.7	30.687	101.7	51.0	8.6	8.8	38.1	8.1	2.1	13.1	1.9	14.2	3.5	11.4	1.7	11.0	2.0	4.4	-10.3	0.2	0.2	0.2	x
PS94-101	7	23	87.4973	179.841	99	-1.45	33.936	0.000	-1.3	3.0	33.577	68.0	33.8	6.5	5.9	26.1	5.4	1.5	8.5	1.3	9.6	2.3	7.6	1.1	7.3	1.3	4.4	-8.3	0.2	0.2	0.2	x
PS94-101	7	14	87.4973	179.841	174.1	-0.07	34.538	0.145	-0.2	1.3	34.415	57.0	25.9	7.0	4.7	20.8	4.2	1.1	6.6	1.0	7.3	1.8	5.9	0.9	5.7	1.0	4.3	-9.2	0.2	0.2	0.3	x
PS94-117	6	11	84.5603	115.996	9.9	-1.73	32.129	-1.886	-3.5	10.0	31.257	97.2	51.7	20.9	8.9	39.1	8.3	2.1	11.3	1.8	13.7	3.1	10.9	1.5	10.0	1.7	4.0	-10.2	0.2	0.2	0.2	x
PS94-117	6	2	84.5603	115.996	50.1	-1.79	33.748	*-1.200*	*-4.9*	6.9	32.454		41.6	12.8	8.1	32.2	7.2	1.9	11.5	1.6	11.7	3.0	9.6	1.5	9.5	1.6	4.3	-9.1	0.3	0.3	0.4	x
PS94-117	4	21	84.5603	115.996	100.2	-0.99	34.347	-0.056	-0.1	1.8	34.894	60.4	27.0	9.2	5.4	22.2	4.9	1.2	7.1	1.2	8.4	2.1	6.7	1.0	6.5	1.1	4.4					
PS94-117	4	15	84.5603	115.996	200.2	1.17	34.836	0.199	-0.3	0.6	34.744	54.5	23.1	7.4	4.1	19.1	3.6	1.0	5.3	0.8	6.5	1.5	5.0	0.7	4.7	0.8	4.0	-10.5	0.2	0.1	0.3	x
PS94-125	7	7	85.0855	139.979	30.3	-1.40	32.107	-0.404	6.2	0.5	33.540	97.0	53.1	12.2	10.0	39.7	9.0	2.3	14.3	2.0	14.1	3.6	11.4	1.7	11.2	2.0	4.1	-10.1	0.2	0.2	0.3	x
PS94-125	7	4	85.0855	139.979	50	-1.47	33.381	*0.072*	*4.3*	-0.6	34.439		42.8	14.1	7.8	35.8	7.2	2.0	13.1	1.6	13.1	2.9	9.5	1.4	9.3	1.6	4.1	-9.1	0.2	0.2	0.3	x
PS94-125	5	16	85.0822	139.773	100.4	-1.31	34.286	-0.213	-0.9	2.1	34.044	65.9	31.3	9.8	5.5	24.3	4.9	1.5	15.6	1.2	8.6	2.1	6.8	1.0	6.5	1.1	4.2	-8.7	0.2	0.2	0.3	x
PS94-125	5	11	85.0855	139.979	200.3	0.31	34.715	0.110	-0.4	0.9	34.592	62.0	22.3	7.2	4.5	19.0	4.2	1.1	6.0	0.9	7.4	1.6	5.9	0.8	5.5	0.9	4.4	-9.4	0.2	0.2	0.3	x

Table S2. Endmember values used for water mass calculation for the Nd-method. Discharge values are taken from R-Arctic NET[33].

	salinity	$\delta^{18}O$ [‰]	Nd [pmol/kg]	Nd [pmol/kg] estuary loss 75%	^{143}Nd [pmol/kg]	^{144}Nd [pmol/kg]	ε_{Nd}	discharge [km^3/year]	References for [Nd] and ε_{Nd}	Reference for $\delta^{18}O$ and salinity
**Atlantic**	35.09	0.3	16		2	4	-11.7		Laukert et al. (2017a)[21]	Bauch et al. (2011)[19]
**Pacific**	32.7	-1.1	30		4	7	-5.5		Porcelli et al. (2009)[34]	Bauch et al. (2011)[19]
**Lena**	0	-20	744	186	23	44	-15.7	485	Laukert et al. (2017b)[23]	Bauch et al. (2011)[19]
**Yenisei**	0	-15.5	154	39	5	9	-5.2	579	Zimmermann et al. (2009)[35]	Dubinina et al. (2017)[36]
**Ob**	0	-15.5	2152	538	66	128	-6.1	394	Zimmermann et al. (2009)[35]	Dubinina et al. (2017)[36]
**Kolyma**	0	-20	129	32	4	8	-6.0	103	Porcelli et al. (2009)[34]	Bauch et al. (2011)[19]
**Yenisei/Ob[1]**	0	-15.5	963	241	29	57	-6.0	973		Dubinina et al. (2017)[36]
**average river[1]**	0	-20	840	210	26	50	-8.7			Bauch et al. (2011)[19]

[1] [Nd] and ε_{Nd} are calculated from the discharge-weighted original endmembers

Table S3. Parameters used for the Nd-method and water mass fractions based on the Nd-method (river gives the sum of Lena and Yenisei/Ob fractions). Endmember values are listed in Table S2. Values in italics are calculated from samples from other (above and below) water depths.

			parameters used for water mass calc				water mass fractions [%] Nd-method				
Station	Cast	water depth [m]	salinity	$\delta^{18}O$ [‰]	Nd [pmol/kg]	ε_{Nd}	Atlantic	Pacific	river	Lena	Yenisei/Ob
PS94-32	9	10	32.784	0.103	16.4	-11.1	92.9	4.9	0.0	0.0	0.0
PS94-32	9	50	34.323	*0.141*	16.3	-11	91.9	6.4	0.0	0.0	0.0
PS94-32	9	101	34.781	0.182	19.9	-11.2	99.6	0.0	1.5	0.5	0.9
PS94-32	9	201	34.985	0.262	20.6	-11.6	100.1	0.0	1.9	1.1	0.8
PS94-40	3	10	33.633	0.085	19.9	-10.6	97.6	1.3	1.3	0.0	1.3
PS94-40	3	30	34.294	*0.013*	*18.8*	-10.9	89.6	9.7	0.5	0.5	0.0
PS94-40	3	50	34.395	*-0.004*	17.7	-10.6	89.4	9.7	0.0	0.0	0.0
PS94-40	3	100	34.562	0.113	20.1	-10.3	93.7	6.0	1.1	0.0	1.1
PS94-40	3	200	34.946	0.285	16.2	-11.1	94.0	5.0	0.0	0.0	0.0
PS94-50	8	10	33.636	-0.114	18.9	-10	82.2	16.7	0.1	0.1	0.0
PS94-50	8	50	34.449	*-0.104*	24.2	-9.8	97.7	0.6	3.2	0.0	3.2
PS94-50	4	200	34.858	0.239	21.6	-10.4	99.9	0.0	2.0	0.0	2.0
PS94-58	5	30	34.235	*0.018*	18.1	-10	82.7	16.2	0.0	0.0	0.0
PS94-58	5	50	34.436	*0.031*	16.9	-10.1	82.4	15.2	0.0	0.0	0.0
PS94-58	3	100	34.576	0.108	18.0	-9.8	80.1	18.7	0.0	0.0	0.0
PS94-58	3	200	34.871	0.209	24.2	-12.5	99.8	0.0	3.8	2.7	1.1
PS94-69	5	10	33.118	-1.169	32.3	-8.2	73.0	21.2	5.8	0.4	5.4
PS94-69	5	25	33.707	*-0.841*	28.3	-8	33.2	64.1	1.9	1.9	0.0
PS94-69	5	50	34.011	*-0.225*	27.4	-8	44.7	53.4	1.9	1.1	0.7
PS94-69	5	100	34.355	0.160	28.9	-8.9	90.3	7.9	4.8	0.0	4.8
PS94-69	4	200	34.829	0.193	24.2	-10.4	99.5	0.0	3.3	0.7	2.7
PS94-81	7	10	30.283	-3.382	47.4	-11.7	30.1	55.9	13.8	13.8	0.0
PS94-81	7	25	32.690	*-2.227*	35.9	-8.4	9.0	84.0	4.9	4.9	0.0
PS94-81	7	50	33.366	*-1.694*	34.7	-7.9	9.5	86.1	3.9	3.9	0.0
PS94-81	5	100	34.070	-0.592	27.2	-8.6	78.4	18.4	3.5	0.0	3.5
PS94-81	5	174	34.585	*-0.015*	26.8	-9.8	98.6	0.0	4.4	0.5	3.9
PS94-96	7	10	28.365	*-3.693*	44.8	-11.9	31.5	48.7	13.4	13.4	0.0
PS94-96	7	30	29.528	*-2.855*	38.1	-12.2	49.7	33.1	10.7	10.7	0.0
PS94-96	7	50	31.736	*-0.587*	33.0	-8.7	16.9	72.0	4.6	4.6	0.0
PS94-96	7	100	33.971	0.061	25.1	-8.5	50.7	47.9	1.2	1.2	0.0
PS94-96	7	200	34.623	0.125	30.8	-9.8	99.0	0.0	6.2	1.3	4.9
PS94-101	9	18	28.132	*-3.779*	30.5	-9.4	27.4	56.3	4.9	4.9	0.0
PS94-101	9	23	28.685	-3.650	33.6	-9.8	24.2	59.8	6.2	6.2	0.0
PS94-101	9	39	29.010	-2.224	32.3	-10	30.8	53.6	6.0	6.0	0.0
PS94-101	9	55	32.122	-0.483	38.1	-10.3	31.3	60.2	7.9	7.9	0.0
PS94-101	7	99	33.936	0.000	26.1	-8.3	70.1	27.9	2.5	0.0	2.5
PS94-101	7	174	34.538	0.145	20.8	-9.2	75.4	24.8	0.3	0.0	0.3
PS94-117	6	10	32.129	-1.886	39.1	-10.2	89.7	0.0	11.0	4.4	6.7
PS94-117	6	50	33.748	*-1.200*	32.2	-9.1	93.0	0.0	7.0	0.6	6.4
PS94-117	4	200	34.836	0.199	19.1	-10.5	92.5	7.7	0.6	0.0	0.6
PS94-125	7	30	32.107	-0.404	39.7	-10.1	96.4	0.0	10.7	4.1	6.6
PS94-125	7	50	33.381	*0.072*	35.8	-9.1	95.7	0.0	8.5	1.2	7.3
PS94-125	5	100	34.286	-0.213	24.3	-8.7	74.9	24.3	1.9	0.0	1.9
PS94-125	5	200	34.715	0.110	19.0	-9.4	76.2	23.2	0.0	0.0	0.0

SI References

1. Cutter, G. *et al.* Sampling and sample-handling protocols for GEOTRACES cruises. GEOTRACES cookbook. (2014). Available at: http://www.geotraces.org/images/stories/documents/intercalibration/Cookbook_v2.pdf. (Accessed: 13th August 2019)
2. Shabani, M. B., Akagi, T. & Masuda, A. Preconcentration of trace rare-earth elements in seawater by complexation with bis(2-ethylhexyl) hydrogen phosphate and 2-ethylhexyl dihydrogen phosphate adsorbed on a C18 cartridge and determination by inductively coupled plasma mass spectrometry. *Anal. Chem.* **64**, 737–743 (1992).
3. Jeandel, C., Thouron, D. & Fieux, M. Concentrations and isotopic compositions of neodymium in the eastern Indian Ocean and Indonesian straits. *Geochim. Cosmochim. Acta* **62**, 2597–2607 (1998).
4. Pin, C. & Zalduegui, J. F. S. Sequential separation of light rare-earth elements, thorium and uranium by miniaturized extraction chromatography: Application to isotopic analyses of silicate rocks. *Anal. Chim. Acta* **339**, 79–89 (1997).
5. Jacobsen, S. B. & Wasserburg, G. J. Sm-Nd evolution of chondrites. *Earth Planet. Sci. Lett.* **50**, 139–155 (1980).
6. O´Nions, R. K., Hamilton, P. J. & Evensen, N. M. Variations in 143Nd/144Nd and 87Sr/86Sr ratios in oceanic basalts. *Earth Planet. Sci. Lett.* **34**, 13–22 (1977).
7. Vance, D. & Thirlwall, M. An assessment of mass discrimination in MC-ICPMS using Nd isotopes. *Chem. Geol.* **185**, 227–240 (2002).
8. Tanaka, T. *et al.* JNdi-1: A neodymium isotopic reference in consistency with LaJolla neodymium. *Chem. Geol.* **168**, 279–281 (2000).
9. Behrens, M. K. *et al.* Rapid and precise analysis of rare earth elements in small volumes of seawater - Method and intercomparison. *Mar. Chem.* **186**, 110–120 (2016).
10. Martin, E. E. *et al.* Extraction of Nd isotopes from bulk deep sea sediments for paleoceanographic studies on Cenozoic time scales. *Chem. Geol.* **269**, 414–431 (2010).

11. Rudnick, R. L. & Gao, S. Composition of the Continental Crust. in *Treatise on Geochemistry* (eds. Holland, H. D. & Turekian, K. K.) (Elsevier, 2003). doi:http://dx.doi.org/10.1016/B0-08-043751-6/03016-4
12. Behrens, M. K., Pahnke, K., Schnetger, B. & Brumsack, H. J. Sources and processes affecting the distribution of dissolved Nd isotopes and concentrations in the West Pacific. *Geochim. Cosmochim. Acta* **222**, 508–534 (2018).
13. Charette, M. A. *et al.* The Transpolar Drift as a Source of Riverine and Shelf-Derived Trace Elements to the Central Arctic Ocean. *J. Geophys. Res. Ocean.* **125**, (2020).
14. Östlund, H. G. & Hut, G. Arctic Ocean water mass balance from isotope data. *J. Geophys. Res.* **89**, 6373–6381 (1984).
15. Alkire, M. B., Morison, J. & Andersen, R. Variability in the meteoric water, sea-ice melt, and Pacific water contributions to the central Arctic Ocean, 2000–2014. *J. Geophys. Res. Ocean.* **120**, 1573–1598 (2015).
16. Jones, E. P., Anderson, L. G., Swift, J. H., Diego, S. & Jolla, L. Distribution of Atlantic and Pacific waters in the upper Arctic. *Geophys. Res. Lett.* **25**, 765–768 (1998).
17. Ekwurzel, B., Schlosser, P., Mortlock, R. A. & Fairbanks, R. G. River runoff, sea ice meltwater, and Pacific water distribution and mean residence times in the Arctic Ocean. *J. Geophys. Res.* **106**, 9075–9092 (2001).
18. Yamamoto-Kawai, M., McLaughlin, F. A., Carmack, E. C., Nishino, S. & Shimada, K. Freshwater budget of the Canada Basin, Arctic Ocean, from salinity, δ18O, and nutrients. *J. Geophys. Res. Ocean.* **113**, 1–12 (2008).
19. Bauch, D. *et al.* Origin of freshwater and polynya water in the Arctic Ocean halocline in summer 2007. *Prog. Oceanogr.* **91**, 482–495 (2011).
20. Anderson, L. G. *et al.* Source and formation of the upper halocline of the Arctic Ocean. *J. Geophys. Res. Ocean.* **118**, 410–421 (2013).
21. Laukert, G. *et al.* Ocean circulation and freshwater pathways in the Arctic Mediterranean based on a combined Nd isotope, REE and oxygen isotope section across Fram Strait. *Geochim. Cosmochim. Acta* **202**, 285–309 (2017).
22. Laukert, G. *et al.* Pathways of Siberian freshwater and sea ice in the Arctic Ocean

traced with radiogenic neodymium isotopes and rare earth elements. *Polarforschung* **87**, 3–13 (2017).

23. Laukert, G. *et al.* Transport and transformation of riverine neodymium isotope and rare earth element signatures in high latitude estuaries: A case study from the Laptev Sea. *Earth Planet. Sci. Lett.* **477**, 205–217 (2017).
24. Rosén, P.-O. *et al.* Ice export from the Laptev and East Siberian Sea derived from δ^{18}O values. *J. Geophys. Res. Ocean.* **120**, 5997–6007 (2015).
25. Charette, M. A. *et al.* Coastal ocean and shelf-sea biogeochemical cycling of trace elements and isotopes : lessons learned from GEOTRACES. (2016).
26. Laukert, G. *et al.* Water mass transformation in the Barents Sea inferred from radiogenic neodymium isotopes, rare earth elements and stable oxygen isotopes. *Chem. Geol.* **511**, 416–430 (2019).
27. Goldstein, S. L. & Hemming, S. R. Long-lived Isotopic Tracers in Paleoceanography and Ice Sheet Dynamics. *Treatise on Geochemistry* 453–489 (2003).
28. Lambelet, M. *et al.* Neodymium isotopic composition and concentration in the western North Atlantic Ocean: Results from the GEOTRACES GA02 section. *Geochim. Cosmochim. Acta* **177**, 1–29 (2016).
29. Zheng, X. Y., Plancherel, Y., Saito, M. A., Scott, P. M. & Henderson, G. M. Rare earth elements (REEs) in the tropical South Atlantic and quantitative deconvolution of their non-conservative behavior. *Geochim. Cosmochim. Acta* **177**, 217–237 (2016).
30. Williford, T. *et al.* Insights into the origins, molecular characteristics and distribution of iron-binding ligands in the Arctic Ocean. *Mar. Chem.* **231**, 103936 (2021).
31. Schlitzer, R. Ocean Data View. (2020).
32. Rabe, B. *et al.* Physical oceanography during POLARSTERN cruise PS94 (ARK-XXIX/3). (2016). doi:10.1594/PANGAEA.859558
33. R-ArcticNET. Available at: http://www.r-arcticnet.sr.unh.edu/v4.0/index.html. (Accessed: 13th August 2019)
34. Porcelli, D. *et al.* The distribution of neodymium isotopes in Arctic Ocean basins.

Geochim. Cosmochim. Acta **73**, 2645–2659 (2009).

35. Zimmermann, B. *et al.* Hafnium isotopes in Arctic Ocean water. *Geochim. Cosmochim. Acta* **73**, 3218–3233 (2009).

36. Dubinina, E. O., Kossova, S. A., Miroshnikov, A. Y. & Kokryatskaya, N. M. Isotope (δD, $\delta^{18}O$) systematics in waters of the Russian Arctic seas. *Geochemistry International* **55**, 1022–1032 (2017).
